# Machine Learning Assists in the Design and Application of Microneedles

**DOI:** 10.3390/biomimetics9080469

**Published:** 2024-08-02

**Authors:** Wenqing He, Suixiu Kong, Rumin Lin, Yuanting Xie, Shanshan Zheng, Ziyu Yin, Xin Huang, Lei Su, Xueji Zhang

**Affiliations:** 1 Guangdong Laboratory of Artificial Intelligence and Digital Economy (SZ), Shenzhen 518000, China; hewenqing@gml.ac.cn (W.H.);; 2 School of Biomedical Engineering, Marshall Laboratory of Biomedical Engineering, Shenzhen University Medical School, Shenzhen University, Shenzhen 518060, China; 3 Institute of Chemical Materials, China Academy of Engineering Physics, Mianyang 621900, China; 4 Shenzhen Key Laboratory of Nano-Biosensing Technology, Marshall Laboratory of Biomedical Engineering, International Health Science Innovation Center, Shenzhen University Medical School, Shenzhen University, Shenzhen 518060, China

**Keywords:** microneedle, machine learning, design principle, application, disease treatment, sensing

## Abstract

Microneedles (MNs), characterized by their micron-sized sharp tips, can painlessly penetrate the skin and have shown significant potential in disease treatment and biosensing. With the development of artificial intelligence (AI), the design and application of MNs have experienced substantial innovation aided by machine learning (ML). This review begins with a brief introduction to the concept of ML and its current stage of development. Subsequently, the design principles and fabrication methods of MNs are explored, demonstrating the critical role of ML in optimizing their design and preparation. Integration between ML and the applications of MNs in therapy and sensing were further discussed. Finally, we outline the challenges and prospects of machine learning-assisted MN technology, aiming to advance its practical application and development in the field of smart diagnosis and treatment.

## 1. Introduction

Microneedles (MNs), as a type of miniaturized medical devices, usually consist of micron-sized needles with heights less than 1000 μm and tip diameters no more than 25 μm [1,2]. They were initially developed as painless and minimally invasive drug delivery devices as they could penetrate the stratum corneum and generate channels for drug diffusion without further touching the nerves and blood capillaries [3]. Nowadays, MNs have been extended to different applications, such as drug delivery [4], skin diseases [5], wound management [6], other organ diseases [7], and biosensing [1,8].

Machine learning, a subset of AI, utilizes computer algorithms to automatically analyze and learn patterns from data, enabling the automatic acquisition of new knowledge and capabilities. It can process a large amount of complex data, extract useful information, make decisions, and improve their performance over time based on experience.

Numerous reviews have examined MNs from various viewpoints of preparation methods and applications. However, to our knowledge, there is no review concentrated on the integration between ML technologies and MN platforms. Here, this article focuses on the design and application of MNs enhanced by ML techniques. Specifically, we introduce the conception and classification of ML and its supportive role in the design as well as disease treatment and sensing applications of MNs. Finally, the prospects of ML-assisted MN development were illustrated to promote the design and extend the application of MNs.

## 2. Introduction of Machine Learning

ML plays a crucial role in advancing biomedical engineering, particularly through its contributions to design optimization [9], material selection [10], drug formulation [11], personalized healthcare [12], and treatment monitoring and feedback [13]. Machine learning algorithms optimize medical equipment design and selection, enhancing functionality and safety and accelerating new product development. In drug formulation, machine learning predicts drug behaviors, optimizes efficacy and safety, and significantly boosts the efficiency of drug development processes. Personalized healthcare benefits from machine learning by customizing treatment plans based on the analysis of genetic information and physiological data, thereby enhancing treatment accuracy and reducing side effects. Additionally, machine learning can monitor treatments in real time, adjust strategies based on feedback, and ensure optimal patient outcomes. These applications not only improve treatment efficiency and accuracy but also enhance patient experiences and outcomes, fostering ongoing innovation in medical technology.

ML is categorized into traditional machine learning and deep learning [14], each possessing distinct characteristics and specific scenarios. Traditional machine learning algorithms rely on scientists to understand the data’s structure and create features that enhance algorithm performance, which are more suited to dealing with limited data and simple problems. In contrast, deep learning utilizes deep artificial neural networks to process large datasets. It is highly effective in automatically learning useful features from data, making it ideal for handling complex data types including images, speech, and text, where features are difficult to extract manually.

### 2.1. The Rise and Development of Supervised Learning

**Figure 1:**
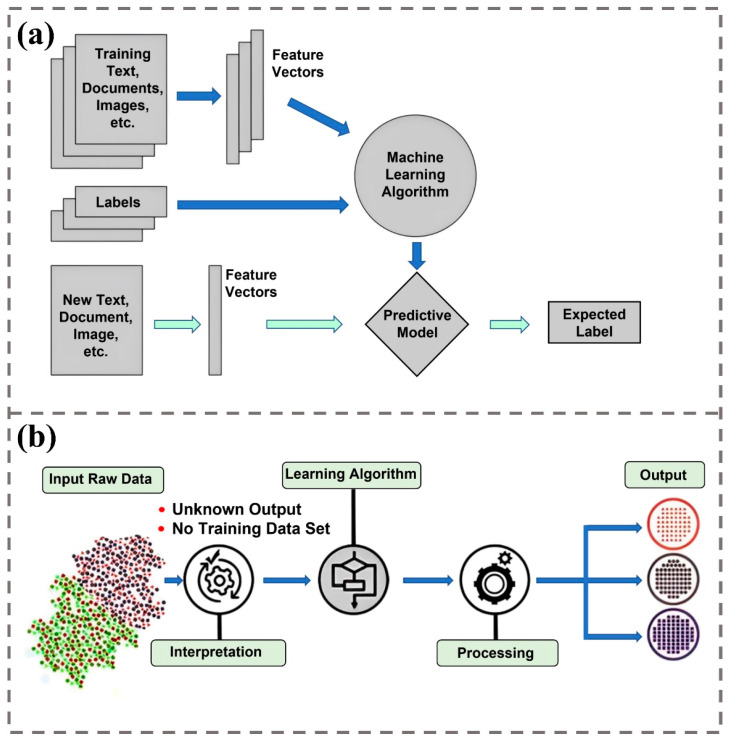
Overview of ML approaches. (**a**) Supervised learning: This algorithm uses training data (texts, documents, images) with known labels to extract feature vectors [21]. These features are then fed into a machine learning algorithm to develop a predictive model. (**b**) Unsupervised learning: This algorithm autonomously identifies patterns and relationships in data without predefined labels [22]. It interprets and processes data without relying on a pre-existing training dataset, often resulting in categorized or structured output.

Supervised learning is one of the earliest and most prevalent forms of machine learning [15]. It fundamentally involves learning the relationship between input data and corresponding output labels (Figure 1a). Typically, the input dataset is divided into a training dataset and a testing dataset. The training dataset consists of samples with known outputs, which the algorithm uses to build predictive models, while the testing dataset is used to evaluate the model’s performance. These models are continuously refined through iterative training, enhancing their ability to accurately predict new, unseen data. The initial method developed for supervised learning is linear regression, which aims to define the optimal linear relationship between data points. A significant advancement in supervised learning was the introduction of the support vector machine (SVM) by Vapnik and others in 1995 [16]. SVMs are adept at both classification and regression tasks. They excel in linear classification and effectively perform nonlinear classification using the kernel trick, which maps inputs into a high-dimensional feature space. Additionally, decision trees and random forests play critical roles in supervised learning. These algorithms make decisions and classifications by constructing tree structures. Random forests, systematically described by Breiman et al. in 2001 [17], are ensembles of decision trees. Each tree makes decisions based on the best feature selected from a random subset of features, enhancing the model’s predictive accuracy and stability by aggregating the predictions of various trees. For classification tasks, the majority voting method is employed, while for regression tasks, the average of the predictions is calculated.

### 2.2. The Rise and Development of Unsupervised Learning

Unsupervised learning distinguishes itself from supervised learning by operating without predefined ground truths or explicit guidance [18]. In this learning paradigm, algorithms autonomously explore the data to identify underlying structures and patterns, using latent features to classify new data. They detect latent features by analyzing datasets and use these learned features to classify new data (Figure 1b). Unsupervised learning is predominantly used in dimensionality reduction and clustering analysis, where it excels in discovering the inherent organization within the data. One of the seminal techniques in unsupervised learning for dimensionality reduction is principal component analysis (PCA), introduced by Hotelling in 1933 [19]. PCA reduces the dimensionality of data by standardizing it, computing the covariance matrix, and then solving for eigenvalues and eigenvectors to identify the principal components. These components are selected based on their ability to explain the maximum variance within the data. PCA is extensively utilized across various fields for data visualization, noise reduction, and feature extraction, making it an invaluable tool in statistical data analysis. Clustering is another fundamental aspect of unsupervised learning, with the K-means algorithm being one of its most prominent methods. Developed by MacQueen in 1967 [20], K-means aims to divide data points into K distinct clusters by maximizing the similarity within each cluster and minimizing the similarity between different clusters. Renowned for its simplicity and effectiveness, particularly with large datasets, K-means finds widespread applications in market segmentation, social network analysis, image segmentation, and document clustering. This algorithm aids in deciphering the intrinsic structure of data, helping users identify and analyze groups of data points that share similar characteristics.

### 2.3. The Revolution of Deep Learning

Deep learning extends the concept of artificial neural networks (ANNs) by employing artificial neurons that function similarly to biological neurons, using nonlinear activation functions to process and transmit input signals. The foundational theory of neural networks advanced significantly with the introduction of backpropagation by Rumelhart et al. in 1986 [23], marking a pivotal moment in the evolution of machine learning. This development enabled effective training of multilayer neural networks, which was not feasible before due to the lack of robust algorithmic support for learning in deep network architectures. Following the proposal of the backpropagation algorithm, several landmark neural network architectures emerged, including LeNet, developed by LeCun et al. in 1989 [24], and long short-term memory (LSTM) networks, introduced by Hochreiter and Schmidhuber in 1997 [25]. Despite these significant advancements in neural network applications, development during this period was still constrained by vanishing or exploding gradients, lack of large labeled datasets, and insufficient hardware capabilities. However, a major turning point occurred in 2006 when Hinton introduced a new concept and training strategy [26] for deep learning, making the training of deeper neural networks feasible. A landmark moment for deep learning came in 2012 when Alex Krizhevsky [27] and colleagues won the ImageNet competition using convolutional neural networks (CNNs), ushering in a new era of machine learning applications.

**Figure 2:**
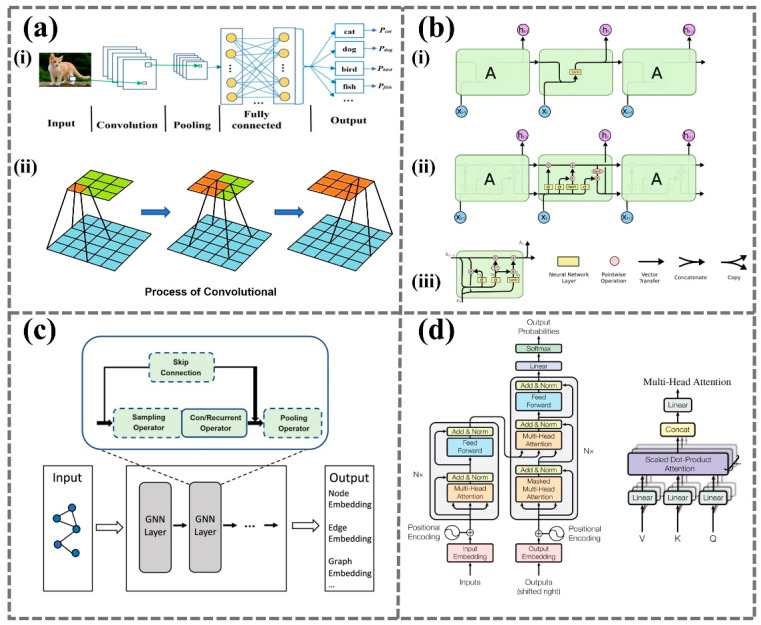
Four types of neural network architectures. (**a**) Convolutional neural network: (i) the model’s structure includes convolutional layers, pooling layers, and fully connected layers to extract features and classify images, (ii) illustration of the convolution process [37]. (**b**) Recurrent neural network (i) and its variants long short-term memory (ii) and gated recurrent unit (iii) process data through recurrent data flow and multi-layer network structures [38]. (**c**) Graph neural network processes node and edge data using graph-based neural networks to generate embedding vectors [39]. (**d**) Transformer is characterized by its multi-head attention mechanism [33].

CNNs use convolutional layers, activation functions, pooling layers, fully connected layers, and output layers to process data in a hierarchical manner (Figure 2a). Each layer builds on features extracted by the previous layer, forming increasingly complex representations. This approach is highly effective for image recognition and classification, making CNNs excel in handling high-dimensional data across various applications including image and video recognition, recommendation systems, and natural language processing (NLP). Yann LeCun [24] and colleagues pioneered the use of CNNs with the development of LeNet-5, a network primarily aimed at handwritten digit recognition. This network marked the beginning of modern CNNs and was successfully implemented in the United States Postal Service’s automatic zip code recognition system. Subsequent developments in CNN architecture, such as the design of VGGNet by Karen Simonyan and Andrew Zisserman [28], demonstrated that increasing network depth can significantly improve performance. Their model featured smaller convolutional windows but a deeper architecture, further enhancing the network’s ability to learn image features.

Recurrent neural networks (RNNs) are optimized for processing sequential data, such as time series analysis, speech recognition, and language modeling. The defining feature of RNNs is their internal state loop, which acts as a form of memory, allowing the network to maintain temporal dynamics and process sequences in context. This feedback structure enables RNNs to preserve the continuity and relevance of information across sequential data, making them particularly useful for tasks that require understanding over time. The introduction of architectures like LSTM networks and gated recurrent units (GRUs) [29] has addressed the challenges of vanishing and exploding gradients that traditional RNNs often faced with long sequences (Figure 2b). These enhancements allow RNNs to learn dependencies over extended sequences, significantly improving their efficacy in complex sequential tasks such as machine translation, text generation, and speech-to-text conversion. LSTM networks and GRUs maintain a balance between learning long-term dependencies and adapting to new data, making them robust choices for advanced sequence modeling.

Graph neural networks (GNNs) are specialized neural networks crafted to process graph-structured data, effectively capturing the intricate relationships and interactions among entities within graphs. GNNs typically include convolutional or recurrent operators (Figure 2c), which are core components used for updating node features and aggregating information. The concept of GNNs was first introduced by Scarselli et al. in 2009 [30], who developed a neural network framework designed specifically for learning directly from graph data. This pioneering effort laid the groundwork for further developments in the field of GNN research. Building upon Scarselli’s foundational work, in 2013, Bruna et al. introduced graph convolutional networks (GCNs) using spectral methods [31]. This innovation marked a significant advancement in the application of deep learning techniques to graph data, providing more efficient and scalable methods for handling complex graph-structured information. A notable development in GNN architecture occurred in 2018 when Veličković et al. introduced the graph attention network (GAT) [32]. The core innovation of GAT is its use of an attention mechanism to dynamically assign weights to the information transmitted between nodes, handling the heterogeneity and complexity of graphs. GATs effectively manage irregular and feature-rich graph data and are widely used in node classification, graph classification, and link prediction. They adapt to the specific characteristics of nodes and edges, making them particularly suitable for complex, irregular graph data.

The Transformer model, introduced by Vaswani et al. in 2017 [33], features a unique structure composed of an encoder and a decoder. Each component consists of multiple layers, each with a self-attention mechanism and a feedforward fully connected layer. The self-attention mechanism dynamically weighs the importance of different words in a sequence, while the multi-head attention mechanism enhances this capability, allowing the model to operate in different representation subspaces simultaneously (Figure 2d). Transformers are primarily used in natural language processing and are particularly suited for tasks requiring a deep understanding of context, such as machine translation, text summarization, and question-answering systems. They address the issues of gradient vanishing and computational inefficiency when processing long sequences, bringing revolutionary changes to NLP and having a profound impact on the broader field of deep learning. In 2019, the advent of multi-modal Transformers like ViLBERT [34] and VisualBERT [35] extended the Transformer architecture to handle both text and image data in a unified model. These developments have been pivotal for tasks such as image captioning and visual question answering (VQA), significantly enhancing the performance of cross-modal understanding and reasoning. Another notable advancement came in 2021 with the introduction of the Switch Transformer by Google [36], which integrates a mixture of experts (MoEs) system. This model employs a dynamic routing mechanism to assign inputs to the most appropriate expert network for processing, thereby optimizing parameter efficiency and computational resources. The Switch Transformer facilitates the training of models with up to trillions of parameters without a proportional increase in computational costs, pushing the boundaries of what is possible with ultra-large-scale models. This technique not only boosts training efficiency but also expands the horizons for research into massive-scale models.

## 3. Machine Learning Assists in the Design of Microneedles

This section mainly discusses the effects of MN dimension, structure, material selection, and manufacturing methods.

### 3.1. Microneedle Dimension

**Figure 3:**
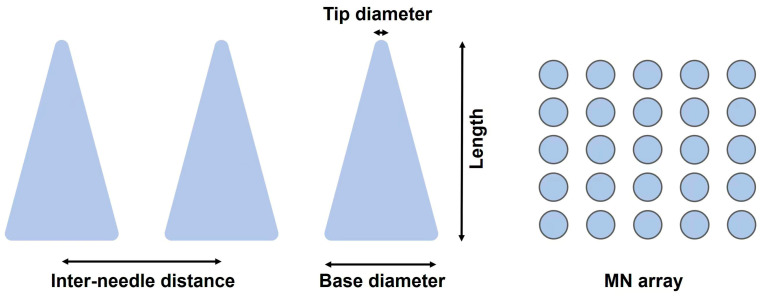
MN dimensions.

The dimensions of MNs, such as needle length, tip diameter, base diameter, and inter-needle distance (Figure 3) significantly influence the penetration performance of MNs and patient comfort. The optimal length of MNs is determined by the depth of the target skin layer. Human skin comprises three layers: the outermost stratum corneum (approximately 10–20 μm thick) [40], the living epidermis (approximately 100–150 μm thick and devoid of blood vessels) [41], and the dermis, which typically measures 3–5 mm in thickness and contains capillaries, lymphatic networks, nerve endings, and sweat glands [42]. Effective drug delivery to the epidermis and dermis requires MNs to penetrate depths greater than 20 μm and 150 μm, respectively. As MN length increases, so does the likelihood of activating pain receptors, due to the dense distribution of these receptors in the deeper dermal layer. Studies indicate a significant increase in pain as MN length increases from 480 μm to 1450 μm, with reported pain rising from 5% to 37%, more than a sevenfold increase [43].

**Figure 4:**
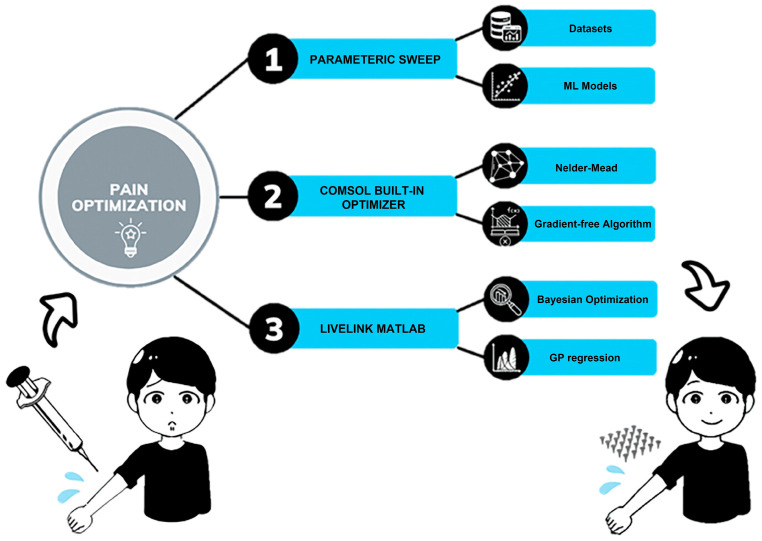
Workflow of three different approaches for pain optimization [44]. (1) Parametric sweep with regression models; (2) COMSOL built-in optimizer; (3) Bayesian optimization using LiveLink.

To minimize pain associated with MN designs with the help of machine learning and optimization tools, one study [44] presented three different approaches and compared their advantages and disadvantages in terms of accuracy, time, and storage space (Figure 4). The workflow of three approaches was illustrated in Figure 4: The first approach involves creating a large dataset through finite element analysis using COMSOL to evaluate pain, followed by using machine learning in MATLAB to train the dataset in order to obtain the optimal regression model. The second approach utilizes the built-in nonlinear optimizer in COMSOL, configured with a gradient-free algorithm, which can accurately formulate optimization problems to minimize pain and find the best MN design. The third approach leverages the LiveLink interface between COMSOL and MATLAB, incorporating Bayesian optimization to explore optimal MN designs, aiming to minimize pain across different age groups.

These approaches select the MN’s inlet diameter, outer diameter, length, thickness, and applied force as input variables, with pain level as the output, evaluating the model’s performance through mean squared error (MSE) and coefficient of determination ($R^{2}$). The final comparison of the approaches, based on the pain index, shows that the third approach offers advantages in efficiency, practicality, and time-saving, making it the preferred choice for tackling complex optimization problems.

**Figure 5:**
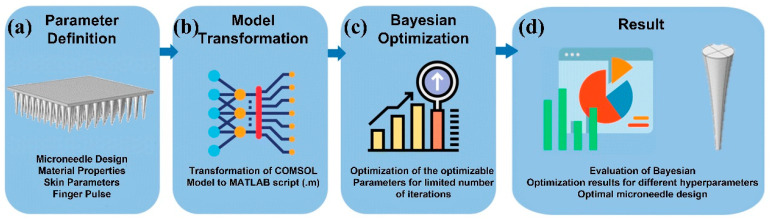
Optimization workflow for MN design [46]. (**a**) Defining key parameters that affect the overall performance of MNs, such as their length, inlet diameter, outlet diameter, wall thickness, and Bessel curve parameters. (**b**) Converting the MN model created in COMSOL Multiphysics into a MATLAB script (.m file). This process takes design parameters as inputs and outputs the volumetric flow rate (VFR), laying the groundwork for subsequent optimization algorithms. (**c**) Applying Bayesian optimization, this algorithm uses the model function as the objective function to systematically adjust parameters and explore through a set number of iterations, aiming to discover parameter combinations that maximize the VFR. (**d**) Assessing the effects of different hyperparameter combinations in Bayesian optimization and identifying the optimal parameter set to achieve the maximum VFR.

The tip diameter of MN is usually determined by factors such as required strength and stability, drug delivery capacity, and patient comfort [45]. Smaller diameter MNs may be more prone to breakage when penetrating the skin, especially when MNs need to penetrate thicker stratum corneum or are used on harder skin areas. Therefore, it is necessary to choose an appropriate diameter to ensure that the MN does not break during use. The diameter of MNs also affects the amount of drug that can be loaded inside or on their surface. MNs with larger diameters may have a larger drug loading space, making them suitable for treatments that require high-dose delivery. Recently, some researchers have combined finite element analysis and machine learning models to determine the optimal physical parameters for MN designs, thereby maximizing the volume flow rate of interstitial fluid (ISF) [46]. Specifically, they model the fluid behavior in MN patches using COMSOL Multiphysics, and optimize parameters such as length, inlet diameter, outlet diameter, thickness, and the Bézier curve that defines the concave profile of the MN using MATLAB’s Bayesian optimization algorithm “bayesopt” function (Figure 5). Ultimately, the optimal parameter combination for maximum volumetric flow rate is determined through optimization over a predefined number of iterations.

The base diameter of MNs is crucial in determining their functionality and stability [47]. Larger base diameters are instrumental in enhancing mechanical stability, which is essential for maintaining the structural integrity of the MNs during skin penetration and minimizing breakage. The options for base diameter are inherently constrained by the manufacturing technology used. This limitation directly impacts the spacing between individual needles within an array, which in turn affects the array’s density. A well-calibrated density is critical for ensuring uniform drug delivery and effective skin penetration, while maintaining the structural integrity of the array.

### 3.2. Microneedle Structure

**Figure 6:**
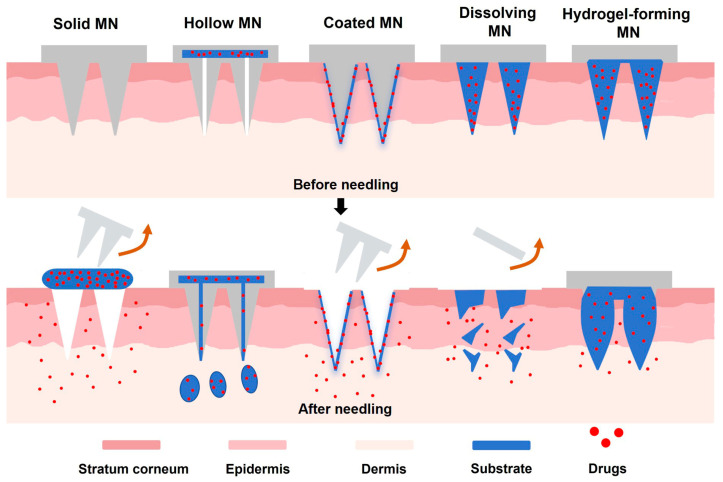
MN structures. Solid MNs, without carrying drugs themselves, deliver drugs through microchannels created as they penetrate the skin. Hollow MNs facilitate direct drug delivery into the skin via their hollow structure. Coated MNs release drugs as their surface coating dissolves during skin penetration. Dissolving MNs gradually melt away into the skin, releasing the drugs. Hydrogel-forming MNs swell upon contact with skin moisture and slowly release drugs.

The structural design of MNs is a critical factor that influences their drug delivery and sampling mechanisms. Currently, the predominant MN can be mainly divided as follows: solid, coated, hollow, dissolved/biodegradable, and hydrogel MNs (Figure 6), each with different drug delivery manners. The initial design of MNs are solid MNs and hollow MNs [48]. Solid MNs are made from durable materials such as metal, silicon, or polymers. They do not directly deliver drugs; instead, their primary function is to penetrate the skin’s stratum corneum to form microchannels. After the MNs are removed, drugs are placed at the puncture sites, allowing the drugs to diffuse and permeate into the skin, thereby accomplishing transdermal drug delivery [49]. Hollow MNs feature a central cavity for direct delivery of drugs or other therapeutic substances to deeper skin layers. This design resembles traditional needle injections but causes minimal pain due to the MNs’ extremely small diameter. However, they require precise manufacturing techniques and can face challenges such as leakage, uncontrolled drug release, or blockage of the narrow channel [50]. Coated MNs have a drug layer coated on the surface. Upon skin penetration, the medication is rapidly released from the MN surface. This design is especially beneficial in scenarios requiring quick drug release, such as pain management and vaccinations. The coatings can be applied through various drug preparation techniques like impregnation, spraying, or spin coating, ensuring uniform and effective drug adhesion [51]. Dissolving MNs are generally composed of polymers, sugars, or other biodegradable materials that dissolve upon contact with ISF after insertion into the skin, thereby gradually releasing drugs into the skin [52]. Hydrogel MNs are made from polymer materials with high water content. These MNs soften and release their contained drugs upon contact with the skin’s moisture. Additionally, hydrogel MNs are designed to avoid sealing off skin pores and can be completely removed from the skin without breaking [53]. The advantages and disadvantages of each MN structure are elaborated in greater detail in other reviews [54].

### 3.3. Material Selection and Fabrication of Microneedles

The selection of materials and manufacturing methods for MNs is crucial in determining their design and application. These factors not only influence the physical and chemical properties of MNs but also affect their biocompatibility, drug release characteristics, and production costs. Six main types of materials have been extensively tested for MN fabrication: silicon, glass, metal, ceramics, sugar, and various polymers. As the first material used to prepare MN [55], silicon has many advantages, such as significant mechanical strength, desirable shapes, and sizes that are flexible for manufacturing. However, the fabricating process is costly and time-consuming. The brittleness of silicon hinders the application of MNs, as they may fracture after insertion and leave silicon residue in the skin. Metals are adopted because of their high fracture toughness and yield strength, making MNs durable and less likely to break when compared with silicon MNs. The first kind of metal material used in manufacturing MNs is stainless steel [56]. However, metal MNs can sometimes cause allergic reactions upon skin penetration. Glass MNs have shown excellent skin penetration performance comparable to silicon MNs, but due to manual processing, their manufacturing time efficiency is relatively low [57]. Ceramics have higher mechanical strength and better resistance to high temperature and humidity [58]. Moreover, the porosity of ceramic needles can be tailored to adjust the drug release kinetics [59]. Polymers offer flexibility, low manufacturing costs, and ease of production. They are predominantly used to make solid, soluble/biodegradable, and hydrogel MNs [60]. Sugar has emerged as a favorable material for MNs preparation due to its natural origin, safety, and rapid solubility [61]. Combining sugar with other polymers enhances the mechanical strength and stability of the MNs.

**Figure 7:**
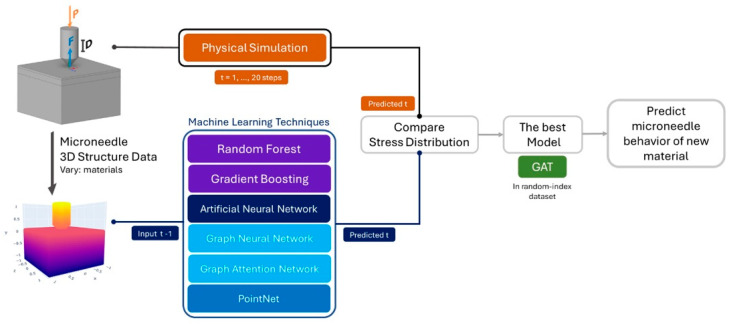
The process of predicting stress distribution in MNs with different materials using physical simulations and machine learning techniques: three-dimensional structural data of stress distribution in MNs made from different materials are generated through physical simulations. Various machine learning techniques (including random forest, gradient boosting, artificial neural network, graph neural network, graph attention network, and PointNet) are used to train on data from time step t − 1 and predict the stress distribution at the next time step t. By comparison, it is determined that the graph attention network (GAT) performs the best [62].

Considering proper MN material selection and fabrication requires sophisticated and costly semiconductor technology, one study [62] used tapered-cone MNs made from 15 different materials to simulate data, creating a dataset with approximately 30,000 mesh elements. Three-dimensional stress and strain data at the MN penetration points were extracted as input datasets, and multiple machine learning models were trained to predict the stress curves of MNs made from different materials, aiming to accelerate the material selection process of MNs. Specifically, the three-dimensional stress data at the tips are taken as input at time step t − 1, and the models then predict the stress distribution for the subsequent time step, t. The predictions were then compared with the simulation data, and the models’ performances were evaluated using MSE. The study explored the performances of random forest (RF), gradient boosting (GBoost), ANN, GNN, GAT, and PointNet models (Figure 7). Ultimately, the study found that the GAT model was the best at predicting the von Mises stress of MNs, with an MSE of 8.3 × 10^−5^ MPa. The final model required only 7 milliseconds to assess a new MN material, dramatically quicker than the actual preparation in the laboratory. This indicates that the GAT model is effective in predicting the performance of new materials, making it a valuable tool for designing and optimizing MN development.

**Figure 8:**
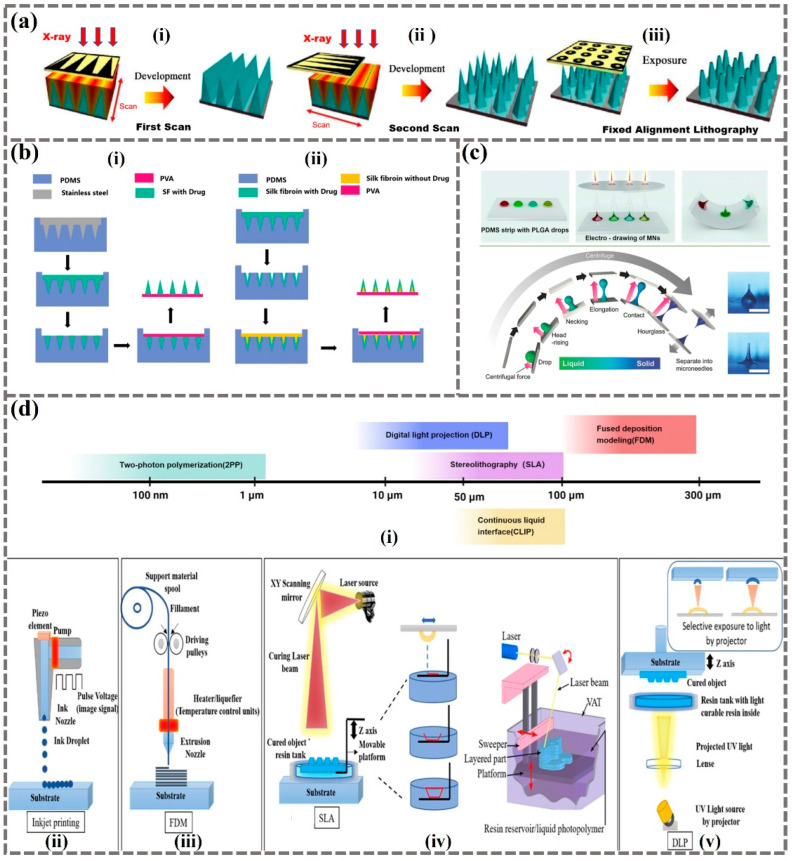
Fabrication of MNs. (**a**) MEMS-based method: twice X-ray lithography process (i,ii) and the fixed alignment for the final exposure (iii) [73]. (**b**) Micromolding method: the DMNs (i) and multilalyered pyramidal microneedles (ii) were prepared with PDMS molds [74]. (**c**) Drawing-based method: electro-drawing process and centrifugal lithography [75,76]. (**d**) 3D-printing-based methods: (i) the resolution ranges of different 3D printing technologies. Schematic diagrams of inkjet printing (ii), fused deposition modeling (iii), stereolithography (iv), and digital light processing (v) [77,78].

Currently, MN manufacturing technology includes microelectromechanical systems (MEMS)-based methods, micromolding, drawing-based methods, and 3D printing-based methods [63]. MEMS technology involves constructing micro-mechanical equipment using integrated circuit manufacturing techniques. In MN fabrication, MEMS is particularly well-suited for creating fine structures like hollow MNs or complex MN arrays. This technology allows for precise control over the size and shape of MNs, ensuring uniformity and functionality. The MEMS manufacturing process typically includes steps such as photolithography (Figure 8a), etching, and shaping, enabling precise manipulation of materials like silicon or metal at extremely small scales. The main advantage of MEMS is the ability to produce MNs with high consistency and complex structures on a large scale, although it comes with high manufacturing costs, especially in the early development stages [64].

Micromolding is the most widely used technology for manufacturing MNs [65], involving techniques like laser drilling, injection molding, or casting using polymer materials (Figure 8b). The process generally starts with creating a micro-mold [66] using precision techniques such as lithography or electron beam machining. Following mold creation, the injection step occurs where the polymer is heated to a flowable state, injected into the mold, and then cooled to solidify, forming the MNs. Micromolding is considered cost-effective, suitable for large-scale production, and capable of rapidly producing MNs with high precision and consistency [67].

Drawing-based methods utilize various types of drawing forces including electrostatic force and centrifugal force (Figure 8c) to form MN-like shapes. Specific curing processes are subsequently applied to solidify and make MNs [68]. These methods are particularly effective for creating MNs with very tiny tips, enabling relatively fast and cost-effective production compared to technology-intensive methods such as MEMS. The simplicity of this approach makes it suitable for producing solid and hollow MNs, with adjustable needle length and sharpness.

As an emerging and accessible technology, 3D printing offers time and cost efficiency in the manufacturing of MNs. Current 3D printing technologies for MNs predominantly use resin photopolymerization, including techniques such as two-photon polymerization (TPP), inkjet printing, fused deposition modeling (FDM), stereolithography (SLA), digital light processing (DLP), projection-based printing (PBP), liquid crystal display (LCD), and continuous liquid interface production (CLIP) (Figure 8d). Notably, TPP is the only method capable of manufacturing nanoscale complex 3D needles with unique structures [69], such as rocket-shaped and mosquito-shaped needles that reduce puncture force, insect-shaped needles for rapid drug/vaccine application, and conical needles for better tissue adherence [70,71]. Despite its high printing resolution, TPP is limited by its low production efficiency, small product size, and high cost. SLA prints in a point-to-point manner with resolutions in the micrometer range, while PBP, LCD, and CLIP utilize layer aggregation methods to solidify patterns layer by layer [72].

Machine learning can use the geometric details of MN and its 3D printing process parameters to identify potential design errors [79], tune the microstructure to achieve desired mechanical properties [80], decrease the energy consumption [81], create 3D printing substitutes to simulate target settings [82], and forecast the drug release of 3D-printed drugs [83].

**Figure 9:**
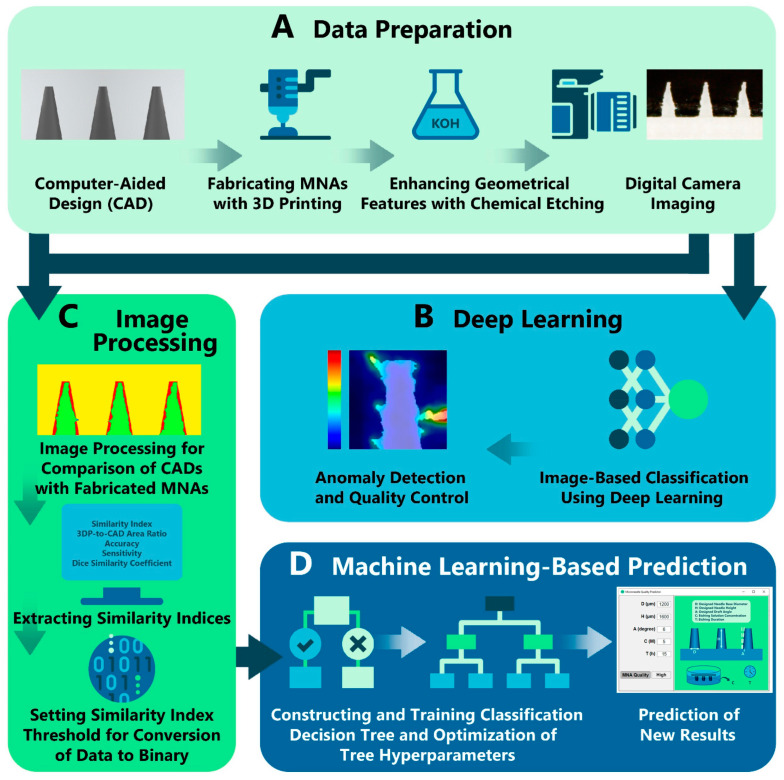
Workflow of preparing and processing data for AI models [84]. (**A**) Manufacturing of MNs and acquisition of MN images. (**B**) Identifying anomalies or defects in images through deep learning models. (**C**) Comparison between CAD images and actual manufactured MN images, extracting similarity indices. (**D**) Optimizing decision tree hyperparameters and applying the trained model to predict outcomes for new data.

One study [84] focused on assessing and predicting 3D-printed MN features through AI frameworks. They drew ten different geometric shapes of MNs using computer-aided design (CAD) and fabricated biodegradable polylactic acid MNs utilizing FDM 3D printing. The 3D-printed MNs were then etched with potassium hydroxide (KOH) solution to enhance their geometric features. Researchers captured 240 images of etched microneedle arrays (MNAs), totaling 2400 MN image samples (Figure 9A), and these data were labeled as “non-defective” or “defective” through expert review. Subsequently, 450 non-defective MN images were selected to train the deep learning model, enabling the analysis of quality control and anomaly detection (Figure 9B). The models tested included ResNet34 [85], MobileNetV2 [86], ConvNeXt_Base [87] based on CNNs. In the experiment of MN anomaly detection, the ConvNeXt_Base model exhibited the best performance with an accuracy of 0.96. Furthermore, to compare the original design with the manufactured MNAs, five parameters (three design features: base diameter, height, and drafting angle of MNs; and two etching features: etching solution concentration and etching duration) were used as input parameters for the machine learning model (Figure 9C). Specifically, a similarity index was calculated by converting CAD images and MN images into binary for pixel comparison. Finally, a classification tree was constructed to find the optimal model configuration, enabling more accurate prediction of MN quality based on the given parameters (Figure 9D).

## 4. Machine Learning Assisted the Application of Microneedles

MNs are characterized by their semi-invasive structures, which allow them to painlessly and precisely target skin and other tissues for accurate drug delivery, sampling, and sensing. Machine learning is a technique that enables computer systems to automatically learn and improve their performance from data through algorithms and statistical models. In the practical application of MNs, a large number of physiological related data will be generated. Through the accurate analysis and learning of machine learning, the application of MNs can be further achieve intelligent upgrading, better meet the development needs of modern society, and expand the breadth and depth of its application. Starting from the application of ML-assisted MNs, we will review and analyze the application examples in this cross-cutting field. At the same time, we will also integrate opinions and views to provide readers with a more comprehensive and in-depth introduction and interpretation.

### 4.1. Application in Treatment

The initial design of MNs was for painless and minimally invasive drug delivery for the treatment of the human body, and drug delivery therapy is the most essential function of MNs. The learning and analysis capabilities of machine learning can enhance the application of MNs in treatment. This section summarizes and reviews the important research achievements in machine learning-assisted MN therapy in recent years.

#### 4.1.1. Treatment with Microneedles

As a novel transdermal drug delivery strategy, MNs can construct a delivery channel that penetrates the skin without touching nerve endings or capillaries, thereby achieving efficient drug delivery and a good user experience [88]. By utilizing the local administration capability of MNs, relatively low doses of drugs are needed to achieve the desired therapeutic effect, thereby reducing the risk of autoimmune dysfunction. MNs have been used in various aspects of treatment, including skin diseases, wound healing, vascular diseases, neurological disorders, diabetes, and arthritis.

1. Skin diseases

**Figure 10:**
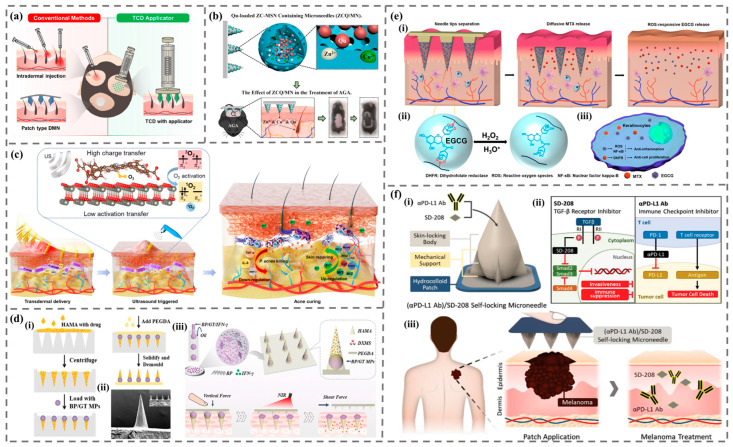
The application of MNs in the treatment of skin diseases. (**a**) A schematic diagram of using a new type of candle light DMN encapsulated with patch free triamcinolone acetonide to treat alopecia areata [93]. (**b**) Schematic diagram of a composite MN patch containing Zn/Cu double-doped mesoporous silica nanoparticles loaded with quercetin for the treatment of AGA [96]. (**c**) Schematic diagram of ultrasound catalytic mechanism and treatment of acne with MN patches based on efficient sonodynamic ion therapy [100]. (**d**) Schematic diagram of detachable MN patches triggered by photothermal responsive microspheres (MPs-MNs) for the treatment of systemic lupus erythematosus [103]. (i) Manufacturing process of MPs-MNs. (ii) SEM images of MPs-MNs; the scale is 200 µm. (iii) Composition and controllable separation mechanism of MPs-MN patches. (**e**) Schematic diagram of the working mechanism of detachable H_2_O_2_-responsive gel-based microneedle patches [104]. (i) After insertion into psoriatic-like skin, the tip of the gel microneedle is separated from the support array to remain in the skin and expand into a porous gel, and then the methotrexate carried is rapidly diffused and released, providing timely treatment for psoriasis. (ii) The release principle of EGCG, triggered by reactive oxygen species and acidic pH. (iii) MTX inhibits the growth of keratinocytes by interfering with dihydrofolate reductase (DHFR) expression, and EGCG clears ROS in psoriatic lesions to prevent the activation of NF-κB inflammatory pathway. (**f**) Schematic diagram of a soluble self-locking microneedle patch for the treatment of skin cancer [108]. (i) The geometric shape of self-locking microneedles. (ii) The mechanism of treating melanoma. (iii) The working mechanism of soluble self-locking microneedle patches.

Skin is the largest organ of the human body as well as the major site where microneedles are applied [89]. At present, MNs are widely used in the treatment of skin diseases such as alopecia areata, hair loss, acne, herpes, systemic lupus erythematosus, psoriasis, and skin cancer (Figure 10).

Alopecia areata is a common skin disease characterized by sudden or gradual shedding of hair, which may be related to genetic, autoimmune, or neuropsychiatric factors. The studies of Chandrashikar et al. [90] and Asad et al. [91] have shown that the transdermal delivery of triamcinolone acetonide through MNs is highly effective in the treatment of alopecia areata. Wei et al. observed the clinical effects of MN administration of minoxidil combined with triamcinolone acetonide in the treatment of alopecia areata [92]. The results showed that for patients with mild alopecia areata, MN administration was significantly more effective than traditional local hormone injection therapy. Lee et al. developed a shooting-type TA-encapsulated candlelit-dissolving microneedle (TCD) utilizing hyaluronic acid and polyvinylpyrrolidone [93]. The TCD applicator ensures uniform insertion and targeted delivery of triamcinolone acetonide to the dermis and hair follicles. The results indicate that TCD using an applicator system is a promising technique for treating alopecia areata (Figure 10a). This treatment method of microneedling is generally suitable for patients with mild to moderate alopecia areata and has the characteristics of fast therapeutic effect and small side effects.

Alopecia is caused by various factors, including age, drugs, and hormones [94]. Yang et al. described a detachable MN patch mediated drug delivery system, primarily composed of hair-derived keratin, for sustained delivery of hair follicle stem cells activators, promoting hair follicle regeneration internally [95]. The results indicate that this MN device, combined with extracellular vesicles derived from mesenchymal stem cells and small molecule drug UK5099, can improve treatment efficiency while reducing dosage. Zhang et al. developed a nanocomposite microneedle (ZCQ/MN) patch containing copper/zinc dual-doped mesoporous silica nanoparticles loaded with quercetin (ZCQ) for the treatment of androgenic alopecia (AGA) [96]. The results showed that the systematic intervention of ZCQ/MN patch on different pathological stages of AGA is an effective hair loss treatment strategy (Figure 10b).

Acne is a common chronic inflammatory disease of the hair follicles and sebaceous glands. Fractional radiofrequency microneedles (FRMs) have been developed as minimally invasive devices for acne treatment and skin regeneration. The device can induce long-term dermal remodeling of the reticular dermis by stimulating the skin, promoting local blood circulation and collagen regeneration. Lee et al. evaluated the therapeutic effect of FRM on Asian patients with acne vulgaris, and the results demonstrated the sebosuppressive effect from a single FRM treatment [97]. In addition, there are various MN patches used to treat acne. For example, Zhang et al. prepared a drug loaded ROS responsive MN patch, which effectively suppressed inflammation induced by Propionibacterium acnes without causing significant side effects [98]. Xing et al. prepared and evaluated a dissolving microneedle (DMN) containing azelaic acid (AZA), and the results showed that AZA-DMN can quickly eliminate acne abscesses and accelerate skin lesion healing [99]. Xiang et al. reported a transdermal delivery of a sodium hyaluronate MN patch mediated by ultrasound responsive nanoparticles, and the results showed that the design of ultrasound-driven MN patches can not only effectively treat acne infection, but also extend to treat other skin wound infections (Figure 10c) [100].

Herpes is a common viral infection caused by the herpes simplex virus, which is spread through direct contact with infected lesions or bodily fluids. Although herpes cannot be cured, antiviral medications such as acyclovir can control symptoms and reduce transmission. Prompt diagnosis and treatment are essential to relieve symptoms and prevent transmission. DMNs loaded with acyclovir were designed and demonstrated superior delivery effects since they can deliver more drugs to the basal epidermis [101]. Nagra et al. evaluated the feasibility and effectiveness of using sterile locally freeze-dried tablets on autolytic MN-treated skin to enhance the skin permeability of acyclovir [102]. This autolytic MN-assisted local chip will be effective for infections in the skin layer and systemic treatment.

Systemic lupus erythematosus is a chronic, diffuse connective tissue disease. Fan et al. designed a controllable and separable MN patch by integrating photothermal responsive phase change microspheres (MPs) into conical hydrogel MNs [103]. Such MPs consist of near-infrared (NIR)-responsive black-phosphorus (BP) and phase-changing gelatin (GT) and are fabricated using a simple capillary microfluidic device (Figure 10d). The intelligent MN patch can effectively load and release two drugs to improve the disease progress of systemic lupus erythematosus treatment.

Psoriasis is a chronic inflammatory skin disease that is generally believed to be related to genetic factors, environmental factors (such as trauma, infection, etc.), and immune factors. Bi et al. have developed detachable H_2_O_2_-responsive gel-based MN patches that are capable of simultaneously delivering the anti-cell proliferation drug methotrexate (MTX) and the reactive oxygen species (ROS) scavenger epigallocatechin gallate (EGCG) [104]. This MN patch can intelligently react with ROS in the skin, ensuring sustained delivery for effective psoriasis treatment (Figure 10e). In addition, Zhang et al. developed a perforated MN, which can promote local T cell migration for the treatment of psoriasis [105].

Skin cancer is the most common malignant tumor worldwide, and researchers have successfully detected nucleic acids (such as pDNA), SiRNA, and other substances that are encapsulated in nanosystems and delivered into the skin using MNs, achieving effective treatment of skin cancer. For example, Hao et al. have created a near-infrared responsive soluble MN that is capable of achieving a single-dose cure for human epidermoid carcinoma and melanoma [106]. In addition, Shao et al. developed a self-heating MN drug-loaded patch with a multi-level structure, which can generate a self-heating effect during administration, promote drug release and penetration, and thus enhance therapeutic efficacy [107]. Joo et al. integrated SD-208 (a novel transforming growth factor-β (TGF-β) inhibitor that inhibits tumor proliferation and metastasis) and anti-PD-L1 (aPD-L1 Ab) (an immune checkpoint inhibitor that induces T cell-mediated tumor cell death) into soluble self-locking MNs, which can effectively treat melanoma (Figure 10e) [108].

2. Wound healing

MNs can painlessly penetrate the stratum corneum and directly transfer protein drugs, nucleic acids, and even cells into the systemic circulation, while maintaining their activity over the whole delivery process [109,110,111]. In addition, compared to traditional detection devices, the microneedles can directly contact the ISF at the wound site, monitoring the environmental changes of the wound. (The biosensing application of MN in wound healing process will be introduced in the next section).

**Figure 11:**
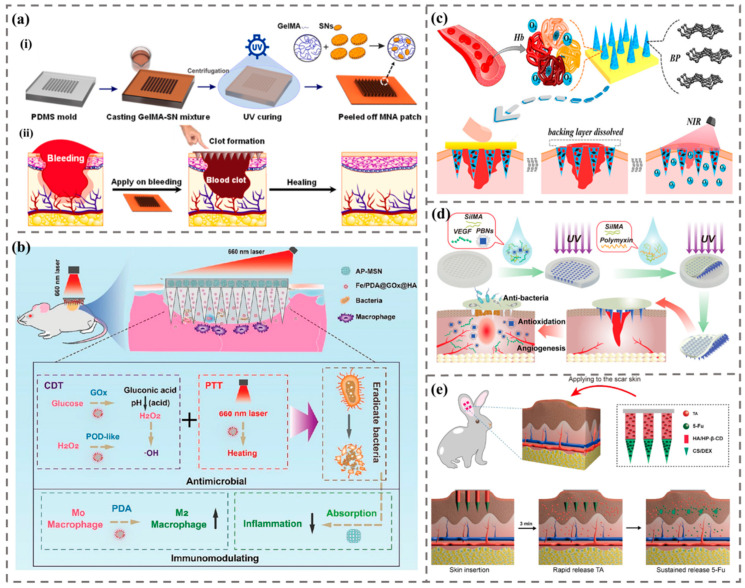
The application of MNs in wound healing. (**a**) Schematic diagram of MN patches applied for rapid hemostasis [115]. (i) Manufacturing of hemostatic MN patches. (ii) The workflow of hemostatic MN patches. (**b**) Schematic diagram of the thermal mechanism of MN with antibacterial and anti-inflammatory properties on wound healing in affected wounds [119]. (**c**) Schematic diagram of a detachable MN loaded with black phosphorus as a responsive oxygen delivery carrier for wound healing [120]. (**d**) Schematic presentation of the fabrication and application of MN-PBNs VEGF patches for promoting diabetic wound healing [124]. (**e**) Schematic diagram of bilayer dissolving MNA therapy applied to the treatment of hypertrophic scars [125].

Wound healing is a dynamic process consisting of four programmed phases: hemostasis, inflammation, proliferation and remodeling (Figure 11) [112,113]. Firstly, it is necessary to quickly stop bleeding and accelerate coagulation. Jeon et al. developed a double-layer adhesive microneedle patch formed by hydrogel [114]. The patch has the core of filamentous protein and the shell of mussel adhesive protein with swelling characteristics, achieving superior tissue adhesion and closure under wet and/or dynamic conditions. Haghniaz et al. prepared bioadhesive MN patches doped with silicate nanosheets (SNs) using gelatin methacryloyl (GelMA) as the matrix material [115]. The MN patch accelerated the in vitro coagulation time from 11.5 min to 1.3 min. In a rat liver hemorrhage model, engineered MNA reduced bleeding by approximately 92% compared to the untreated injury group (Figure 11a).

Secondly, it is necessary to prevent wound inflammation and combat wound infection. For example, Permana et al. used solvent-based MNs to deliver responsive particles with antibacterial effects [116]. In addition, MNs can be used to deliver goods that regulate inflammation. Yao et al. proposed a novel Zn MOF encapsulated MN patch with antibacterial activity and degradability to promote wound healing [117]. Xu et al. encapsulated chloramphenicol in gelatin nanoparticles and polymer matrix to prepare water-soluble polymer MN patches [118]. In addition, MNs can be utilized to deliver cargo that modulates inflammation. Li et al. prepared MN patches with antibacterial and immunomodulatory properties, which included their team making nanoparticles (Fe/PDA@GOx@HA) with antibacterial and pro macrophage M2 transformation properties (Figure 11b) [119]. In addition, the amino functionalized mesoporous silica in the MN base adsorbs pro-inflammatory factors such as free nucleic acids, inhibits sustained inflammatory responses, and promotes the healing of infectious wounds.

Finally, assistance is needed for the growth and repair of newly formed tissues. The main use of MNs can effectively provide live cells and bioactive factors for skin regeneration. Zhang et al. designed separable responsive MNs containing BP, with BP quantum dots and hemoglobin loaded at the tip of the MN, for delivering reactive oxygen under NIR radiation stimulation to promote wound healing (Figure 11c) [120]. Yao et al. developed a phase change MN patch loaded with parathyroid hormone (PTH), which achieved non-invasive sustained release of PTH in rats, promoting wound healing by accelerating extracellular matrix deposition and improving the structure of the extracellular matrix [121]. Long et al. developed MN patches that provide collagen to promote wound healing, treating chronic wounds by anti-inflammatory means and enhancing cell proliferation and angiogenesis [122].

Diabetes is a chronic disease marked by high blood sugar. Since the high glucose environment in the body will affect wound healing, the wound healing of diabetic patients will be more difficult. Yuan et al. developed a methacrylate gelatin MN patch to achieve transdermal and controlled release of secretions and tazarotene and promote wound repair in diabetes [123]. Guan et al. encapsulated Prussian blue nanoenzymes (PBNs) and vascular endothelial growth factor (VEGF) on the tip of MN patch (Figure 11d) [124]. This MN (MN-PBNs-VEGF) patch can promote oxidation, has excellent antibacterial effect, and can achieve transdermal drug delivery for diabetes wound healing.

Additionally, MNs can be used for scar treatment. Scars are a general term for the morphology, appearance, and histopathological changes of normal skin tissue caused by different traumas. MNs stimulate the skin by using many tiny needles to penetrate nutrients into the dermis. Yang et al. fabricated a double-layer DMN array containing 5-fluorouracil and triamcinolone acetonide, which has a biphasic release curve and can provide an excellent alternative strategy for the sustained and minimally invasive clinical treatment of hypertrophic scars (Figure 11e) [125]. MNs can accelerate skin metabolism, promote the reorganization and regeneration of collagen and elastic fibers in the skin, thus reducing local scars.

3. Other diseases

**Figure 12:**
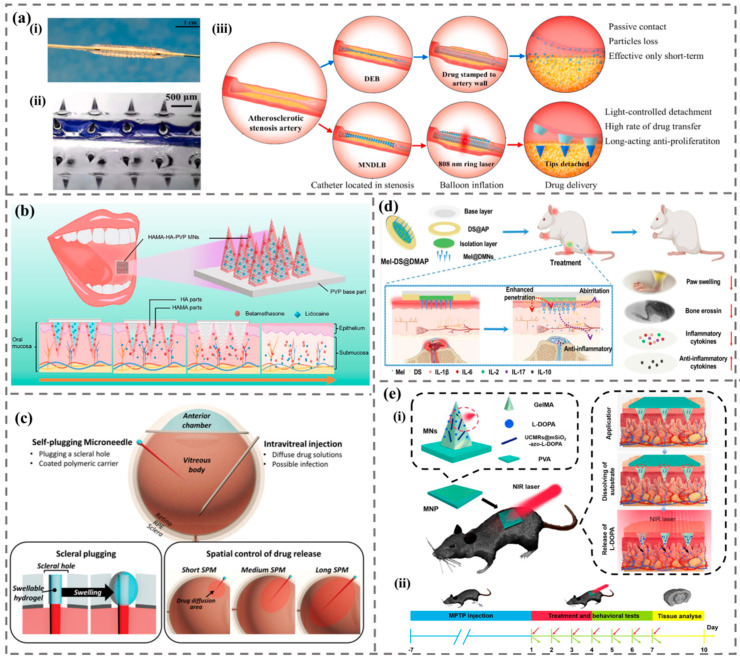
The application of MNs in the treatment of other diseases. (**a**) Schematic diagram of tip-separable microneedles drug-loaded balloon (MNDLB) for the treatment of arteriosclerosis [127]. (i) Dilated MNDLB. (ii) The shape of MNDLB. (iii) Schematic illustration of balloon angioplasty. (**b**) Schematic diagram of a double-layer dissolution MN used for sequential delivery of multiple drugs through the mucosa for the treatment of oral mucosal diseases [130]. (**c**) Schematic diagram of self-plugging microneedles (SPMs) used for intravitreal drug delivery in the treatment of eye diseases, including self-scleral blockage and spatial control of drug release within the vitreous cavity [134]. (**d**) Schematic diagram of a novel composite therapy for rheumatoid arthritis consisting of dissolving microneedles and adhesive transdermal patches [139]. (**e**) Schematic diagram of a molecular motor-based MN patch for the treatment of Parkinson’s disease [141]. (i) The working principle of controlled release MNs containing anti-Parkinson’s drugs. (ii) The treatment plan of this MN for Parkinson’s disease.

MNs are particularly common in the treatment of skin diseases and wound healing due to their targeting ability to work within the skin. Besides, MNs are widely involved in the treatment of other diseases (Figure 12).

At present, the application of MN in the treatment of vascular diseases is mainly reflected in the treatment of intimal hyperplasia and cardiovascular and cerebrovascular diseases. Lee et al. utilized flexible and porous silk fibroin MNs to puncture the outer membrane of blood vessels, continuously and slowly releasing drugs into the middle membrane, effectively inhibiting endothelial proliferation and lesions [126]. Huang et al. developed a drug-loaded balloon with tip detachable MNs on the surface of the balloon (Figure 12a) [127]. Compared with the conventional drug-eluting balloon, it can transfer twice the antiproliferative drugs to the vascular tissue and has excellent cardiovascular and cerebrovascular therapeutic effects.

MNs can directly target the lesion site via the oral mucosa, avoiding the diluting effects of saliva and other factors, thereby enhancing bioavailability and therapeutic efficacy [7]. Li et al. developed a composite MN patch for oral mucosal delivery, which exhibited rapid and effective drug release in the oral mucosa [128]. Guo et al. studied betamethasone soluble MN patches for oral ulcer treatment [129]. Meng et al. designed a novel double-layer dissolving MN for sequential delivery of a variety of drugs through the mucosa to treat oral mucosal diseases (Figure 12b) [130]. Manimaran et al. prepared a dissolvable MN patch containing photosensitizer, which can be effectively used for local administration of photosensitizer for oral cancer phototherapy [131].

The application of MNs in ocular diseases mainly uses MNs to penetrate the barrier of ocular tissues and effectively input drugs into the eyes. Than et al. developed a double-layer detachable MN, which can effectively deliver drugs while being anti-inflammatory [132]. Shi et al. used dissolved MNs to treat fungal keratitis. The therapeutic effect of dissolved MNs was the same as that of matrix injection [133]. Lee et al. developed a self-plugging microneedle (SPM), which can avoid needle retraction and can perform intraocular drug delivery and seal the scleral puncture simultaneously (Figure 12c) [134]. Mahfufah et al. mixed the inclusion complexes of fluconazole into the soluble MN eye patch, which improved the efficiency of fluconazole in the treatment of fungal keratitis [135].

Rheumatoid arthritis (RA) is a systemic autoimmune disease with joint stiffness and swelling, characterized by dysfunctional anti-citrullinated protein antibodies and rheumatoid factors [136]. Du et al. prepared polymer MNs loaded with melittin, which can successfully deliver melittin to the skin and effectively inhibit the progression of RA [137]. Wu et al. developed programmable polymer MNs for combined chemotherapy and antioxidant therapy in rheumatoid arthritis, for transdermal delivery of methotrexate and reactive oxygen species scavengers, to inhibit the progression of RA by combining chemotherapy and antioxidant therapy [138]. Zhen et al. used a combination of dissolution MNs loaded with melittin and adhesive transdermal patches to jointly deliver drugs, and the synergistic effect of the two enhanced the treatment of RA (Figure 12d) [139].

MNs are also widely utilized in the treatment of neurological diseases. Liu et al. developed MNs capable of delivering vascular endothelial growth factor, which can promote angiogenesis and functional recovery after stroke [140]. Zhou et al. used molecular motors as pore inhibitors and gate switches to create MN patches for drugs with controllable time and spatial accuracy triggered by near-infrared light (Figure 12e) [141]. The results showed that the MN patches reduced neuroinflammation and dopaminergic neuron death in the substantia nigra, providing a control strategy for on-demand drug delivery in the treatment of Parkinson’s disease. Li et al. prepared a novel composite delivery system combining PLGA nanocarriers and MN technology for the treatment of neurological diseases [142]. McGuckin et al. developed a polymer MN system aimed at enhancing the transdermal delivery of Pramipexole [143]. The results showed that transdermal drug delivery using MN technology could maintain the delivery of the hydrophilic drug Pramipexole and could be used for the potential treatment of Parkinson’s disease.

#### 4.1.2. Machine Learning-Assisted Microneedle Therapy

**Figure 13:**
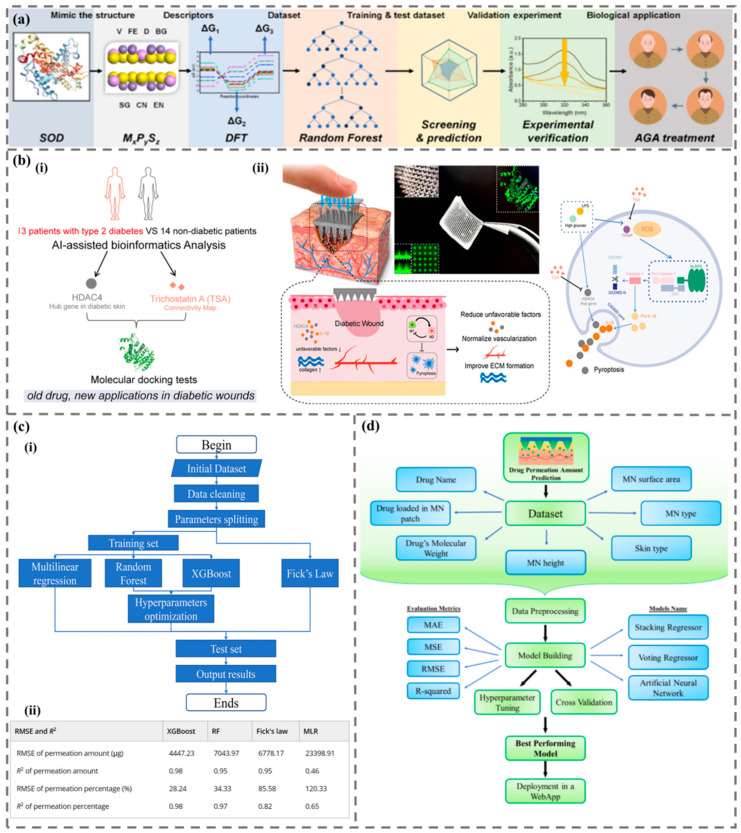
The application of machine learning-assisted MNs in treatment. (**a**) Machine learning guides the discovery of superoxide dismutase nanoenzymes, using MN patches to deliver the goods for the treatment of AGA [144]. (**b**) Machine learning-assisted bioinformatics mining TSA and HDAC4 for diabetes wound healing and combined with MNs to deliver the goods [145]. (i) The discovery of TSA and HDAC4. (ii) The working principle of MN patch. (**c**) Predicting drug penetration through MNs using ML. (i) Schematic diagram of the workflow of the experiment. (ii) Comparison between Fick’s Law and MLR, RF, and XGboost [146]. (**d**) Compare the performance of stacked regressors, artificial neural networks, Voting Regression, and others in predicting the penetration of MN drugs [147].

In summary, MNs are currently widely utilized in various treatments. MN patches are medical devices that deliver drugs or bioactive substances directly to the deeper layers of the skin through tiny needles. The integration of machine learning algorithms opens new avenues for designing and optimizing MN patches (Figure 13).

As evident from the preceding discussion, the potent efficacy of MNs is primarily attributed to their unique transdermal delivery, which overcomes many limitations faced by traditional drug delivery systems. Oral administration methods often suffer from low bioavailability and slow drug absorption. Injection processes require professional handling and can cause discomfort. Traditional transdermal drug delivery methods, such as plasters, ointments, and sprays, are constrained by the skin barrier, resulting in low absorptions and unsatisfactory drug delivery outcomes. In contrast, MNs utilize the concentration gradient between the needles and tissue to release drugs effectively in a multifunctional, efficient, controllable, convenient, safe and comfortable manner. The improvement of MN therapy can be pursued through two main strategies: 1. designing of drugs to be delivered by MNs, and 2. facilitating the penetration and diffusion of therapeutic drugs.

One purpose of machine learning to assist the design of drugs in MNs is to find drugs with better therapeutic effects. Zhang et al. used machine learning to guide the discovery of superoxide dismutase nanozymes for the treatment of AGA [144]. The researchers selected transition metal thiophosphites as potential candidates for nanozymes and tested the machine learning model using 91 different combinations of transition metals, phosphates, and sulfates to select the nanozyme mnps3 with the strongest free radical scavenging ability. The results showed that mnps3 MN patches could achieve higher hair regeneration efficiency at a lower frequency compared with minoxidil, which is widely used at present (Figure 13a). In addition, Xue et al. obtained skin sequencing data of 13 patients with type 2 diabetes and 14 non-diabetic patients from the geo dataset [145]. The therapeutic potential of trichostatin A (TSA) and histone deacetylase 4 (HDAC4) in diabetic wounds was explored by artificial intelligence-assisted bioinformatics analysis (Figure 13b). The results showed that MN-mediated TSA patches could reduce inflammation, promote tissue regeneration, and inhibit HDAC4, thus providing excellent results in diabetic wound healing.

The depth of penetration and the diffusion of drugs are important factors affecting the effect of MN-based therapy. Controlling the depth of drug penetration can reduce the side effects of drugs on normal tissues and improve the treatment efficiency. The introduction of machine learning can avoid in vitro permeation experiments and reduce the experimental cost and time. Yuan et al. used mechanistic models (Fick diffusion law) and statistical models (Multiple Linear Regression (MLR), RF and XGBoost) to predict the penetration of drugs through MN-treated skin membranes, and selected seven features for the machine learning model, including skin type, MN type, MN length, MN surface area, drug loading in MN, drug penetration time, molecular weight of drugs, skin type, and drug penetration time (Figure 13c) [146]. Finally, it was found that the XGBoost model provided the best prediction results. On the other hand, Bisbas et al. compared stacked regression models, artificial neural network models, and voting regression models for predicting drug penetration, and the results showed that the voting regression model performed the best in predicting drug release and its percentage (Figure 13d) [147]. The above indicates that machine learning methods have been found to be useful for predicting skin penetration of drugs treated with MNs.

In addition, MNs have recently been used to discover applications for brain computer interface technology. Brain computer interfaces enable paralyzed patients and people with disabilities to use brain signals to control external devices. The advancement of artificial intelligence represented by machine learning has accelerated the development of the brain computer interface industry. Artificial intelligence is used for pattern recognition in neural signals. Researchers from the University of California, San Diego and Boston University have jointly developed a novel brain computer interface with a scalable and flexible 1024 channel penetrating silicon microneedle array (SiMNA) [148]. SiMNA is the first penetrating MNA with a flexible backing, which allows the device to adhere more evenly to the complex surface of the brain and distribute microneedles piercing the cortex more evenly. A MN that is 10 times thinner than human hair extends from a soft backing, penetrating the surface of brain tissue without penetrating surface small veins and uniformly recording signals from nearby nerve cells in large areas of the cortex.

Indeed, machine learning has emerged as a prominent tool across various domains within biomedical science. Presently, society resides within a digital era where personal information plays a pivotal role. The utilization of machine learning-assisted MN therapy extends beyond the mere above-mentioned applications, encompassing the analysis of individual patient data such as skin type, illness severity, and more to tailor personalized MN treatment plans. Leveraging machine learning models for prediction facilitates the selection of the most suitable MN type, treatment frequency, and medication formula for patients, thereby enhancing treatment efficacy and minimizing side effects. Moreover, machine learning models can forecast future treatment outcomes based on patients’ historical data and ongoing MN therapy, enabling doctors to promptly adjust treatment plans to ensure optimal patient outcomes. Concurrently, machine learning can evaluate the pros and cons of various MN treatment plans, furnishing doctors with decision support. In sum, the integration of machine learning into MN therapy holds vast potential and promising prospects.

### 4.2. Applications in Biosensing

MN biosensing is an innovative method that combines MN technology and biosensing technology. It is used for analyzing or real-time biosensing in situ after sampling and can collect a lot of physiological signals and biomarker data. Machine learning algorithms, such as deep learning and neural networks, can automatically learn and recognize patterns in the data, process and analyze the data, extract useful information, and construct predictive models to diagnose diseases, evaluate treatment effects, and predict disease development. At the same time, it can also optimize the design and performance of MN biosensors. This section summarizes the application of microneedles in biosensing and reviews the important achievements of machine learning-assisted MN biosensors in recent years.

#### 4.2.1. Microneedle Detection Biomarkers

Biomarkers refer to biochemical indicators that can indicate changes or potential changes in the biological system, including organs, tissues, cells, and subcellular systems. Biomarkers are crucial for disease diagnosis, disease staging, and evaluation of the new drugs or therapies. Dermal ISF is defined as the fluid surrounding cells and tissues within the skin, serving as a medium for nutrient reception, waste secretion, and molecular signal communication [149]. Moreover, the ISF is abundant in various biomarkers, enabling effective detection [150].

MNs with few hundreds of microns in height can be applied to the skin in a painless and minimally invasive manner, passing through the stratum corneum and directly entering the dermis ISF to collect biomarkers and metabolites [151]. At present, there are three methods for detecting biomarkers using MNs. The first method is that MNs are only used to collect ISF, and then external instruments are used to analyze the biomarkers in ISF. The second method is to directly capture biomarkers by MNs while collecting ISF, and then analyze the biomarkers they capture. The third approach is to combine MNs with biosensors, collect ISF, and directly analyze it for real-time monitoring.

1. Microneedles for ISF extraction

MN technology utilizes the structure of multi-hollow MNs to absorb liquid through the capillary force. The absorbed liquid is extracted from the skin and stored in MNs or the base layer. The purpose of MNs used for ISF extraction is to extract more ISF in a shorter time. Cheng et al. prepared MNs with excellent swelling ability using biocompatible hydrophilic methacrylic acid hyaluronic acid (MeHA), and the results showed that the MNs extracted approximately 2.3 mg of ISF within 10 min [152]. Zheng et al. made a MeHA-based MN patch, accelerated the extraction rate by osmotic pressure during ISF extraction, and allowed the MN patch to extract 7.90 µL of ISF from pig ear skin ex vivo and 3.82 µL of ISF from mouse back skin in vivo within 3 min [153]. Xie et al. proposed a hollow MN patch integrated with vacuum tubes, which combines the hollow MN patch with a flexible hose to connect the vacuum tubes [154]. Using the negative pressure of a vacuum tube, the MN patch device can effectively collect about 18 μL of ISF from the dermis of living rabbit ears within 5 min. The volume of free ISF that quickly flows into the hose is as high as about 14.80 μL. This hollow MN patch may be currently one of the most efficient minimally invasive devices for in vivo ISF extraction.

2. Microneedles for capturing biomarkers

MNs specially designed for biomarker capture represent a novel approach in modern medical diagnostics [155,156]. In order to make the detection of ISF more effective, a MN patch which can react with biomarkers was designed for capturing biomarkers. Mei et al. designed MNS patches labeled by surface-enhanced Raman scattering tags, densely deposited core-satellite gold nanoparticles, and 3-mercaptophenylboronic acid as Raman reporter genes, making the developed MNs patches highly sensitive and selective in the determination of H_2_O_2_ (an indicator of peritonitis development) for the diagnosis of acute peritonitis [157]. Wang et al. designed bilayer MNs functionalized with biorecognition elements (such as antibodies), thereby enabling selective capture of protein biomarkers in local ISFs in a concentration dependent manner [158]. Yang et al. designed a MNA consisting of a smart DNA circuit hydrogel system encapsulated in hyaluronic acid methacrylate, which can achieve rapid enrichment and sensitive detection of miRNAs in ISF [159].

3. Microneedles combined with biosensors

MNs have great advantages in transdermal sampling. Different from the above, the integration of MNs and biosensors can combine MN sampling and sample analysis, making real-time monitoring and health management possible. Biosensors can transform the interactions of biomolecules into observable signals. In the design of biosensing MN devices, two common strategies are adopted: enhancing the material function of MNs and/or integrating the sensing mechanism into the structure of MN device [8]. Through the optical, electrical, or electrochemical conversion method of the biosensor, the samples sampled by the MN can be observed and analyzed in time to achieve continuous monitoring. The electrode of the MN electronic sensor is usually connected to its source meter through ionic conductive interface or the in situ area filled with conductive liquid or electrolyte. The natural conductivity of the skin can be used to complete the electronic circuit to ensure reliable operation. MN electrochemical sensors are based on MN electronic sensors, which use electrode coating or mixed chemical components to detect biomarkers through potential changes caused by chemical reactions. MN optical sensors use a series of optical signals, such as fluorescence, reflected light, absorbed light, scattering, and interference, to interpret biological data. Different optical sensors include Raman spectrum sensor, colorimetric sensor, and fluorescence sensor.

#### 4.2.2. Machine Learning-Assisted Microneedle Biosensor

**Figure 14:**
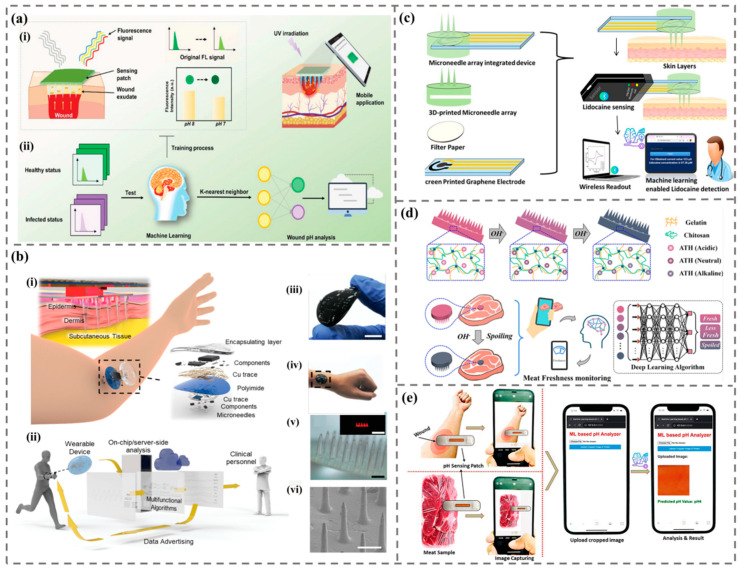
The application of machine learning-assisted MN biosensors. (**a**) Schematic diagram of a multifunctional sensor patch for wound management [163]. (i) The scheme for wound pH monitoring. (ii) Machine learning-assisted pH sensing scheme. (**b**) Schematic diagram of wearable deep tissue oximeter based on MN waveguide [169]. (i) Internal structure of wearable wireless deep-tissue oximeter. (ii) Schematic diagram of intelligent medical management envisaged by sensor patches (iii) Image of the electronic core of sensing patch. (iv) Image of a sensor on human wrist. (v) Image of an array of MN waveguides. (vi) SEM image of the MN waveguides. (**c**) Schematic representation of machine learning-enabled wireless MNA integrated screen-printed electrode-based electrochemical point-of-care device for lidocaine detection [172]. (**d**) Schematic diagram of machine learning-based colorimetric MN sensor for meat freshness monitoring [173]. (**e**) Application process diagram of machine learning-assisted micro needle colorimetric pH sensing patch [174].

Machine learning has the advantages of efficiency, accuracy, predictability, and strong adaptability. It can enhance the practicality of MN biosensors and provide reliable and effective analysis of detection data after MN sensing, making the response of MN sensors more efficient and extensive and enabling them to penetrate the market more effectively. At present, machine learning-assisted MN biosensors are applied in various fields, including wound health monitoring, physiological signal measurement, intracellular drug monitoring, and meat freshness monitoring (Figure 14).

1. Wound health monitoring

Wound infections can result in either mild illnesses or life-threatening diseases, making the monitoring and assessment of the wound healing status critically important [160,161,162]. Traditional monitoring methods are limited by the requirement for sophisticated instruments or skilled operators, and the sampling procedures may cause additional local tissue damage [163]. Therefore, an effective wound management platform is essential to reduce inflammatory complications and infections. MN-based biosensor patches are suitable for wound health monitoring due to their accuracy, efficiency, and low cost and can evaluate the healing degree and health status of wounds.

Under normal circumstances, the skin is weakly acidic (pH, 4–6). However, when a wound is infected, microbial activity creates a toxic, alkaline environment (pH, 7–9) [164,165]. During the process of wound healing, pH is an important parameter value. Shan et al. designed intelligent colorimetric MN patches integrated with Fe ion-gallic acid coordination polymer nanodots [166]. The color of the MN patch changes upon alteration in pH and elevation of H_2_O_2_ concentration. The integrated MNs can catalyze H_2_O_2_ to generate much more OH at the acidic condition for bacteria killing. The results showed that the MN patch can be used for on-demand treatment and real-time reporting of infected wounds. The pH value of the optical MN patch wound needs researchers’ colorimetric observation, which may not be enough in practical application. In order to make the optical MN sensor more intelligent and practical in wound monitoring, machine learning can be introduced.

In 2024, Xiao et al. combined smart MN sensing to inhibit wound infection and track wound healing status (Figure 14a) [163]. The research team encapsulated bi-pcn-222 and curcumin in the tip of the hydrogel MN, made the bottom layer of the MN from polyvinyl alcohol (PVA), and integrated the pH-sensitive fluorescent indicator as the substrate of the MN into the PVA gel to realize the real-time monitoring of the pH value of the wound. Electron transfer from bi-pcn-222 to Staphylococcus aureus will interfere with bacterial metabolism, resulting in a rapid self-disinfection effect. Curcumin has antioxidant and anti-inflammatory effects and can enhance the antioxidant capacity of cells by scavenging reactive oxygen species. The MN patch extracted the wound fluid and delivered it to the PVA gel layer through the tip of Mn and detected the wound pH according to the fluorescence intensity change of the wound pH value of the gel layer. Researchers used smart phones to capture fluorescent images under 365 nm UV light to collect sample data. The KNN model was used to analyze the sensing signal accuracy of 20 sample data, and multivariate classification was carried out for the pH of wound infection. The trained neural network model predicted the pH value more accurately and closer to the experimental value. The results show that the pH-sensitive fluorescent detection patch can be used to evaluate the pH value of the wound, and the prepared MN sensing patch can achieve good wound monitoring.

2. Physiological signal measurement

MN sensors are mainly used to monitor target biomarkers in ISF. At present, MN biosensors are used in various aspects of human biomarker detection for long-term detection and management. Yang et al. developed a wearable continuous blood glucose monitoring (CGM) system based on smartphone control and MNs for long-term blood glucose monitoring [167]. The CGM system consists of three main components: a MN-based glucose sensor, a wearable CGM device, and a customized Android application. Android applications analyze and display detected data in real-time on smartphones. The fully integrated smartphone-based CGM system monitors the daily blood glucose level of diabetes patients, and the data can be further uploaded to the database through the Internet of Things cloud. Goud et al. reported a MN sensing platform for continuous minimally invasive orthogonal electrochemical monitoring of levodopa [168]. The new multimodal MN sensing platform relies on parallel independent enzyme amperometric and non-enzyme voltametric detection of levodopa using different MNs on the same sensor array patch for optimizing drug delivery regimens and effective management of Parkinson’s disease.

In recent years, machine learning combined with MN biosensors has been used for healthcare detection and management. Liu et al. proposed a wireless and wearable deep tissue sensing patch that incorporates biocompatible MN waveguides at the sensing interface, bypassing the light extinction of epidermal and dermal tissues, to achieve tracking of muscle tissue oxygen saturation (Figure 14b) [169]. Sensing patches provide various physiological measurements in the sensing area, including tissue oxygen saturation, pulse oxygen saturation, heart beating, and respiratory activity, and wirelessly transmit data to computing devices for processing. Researchers create simulated datasets by adding Gaussian noise to pure signals (ideal pulse waveforms), and then combine machine learning to analyze and calculate physiological measurement data. The combination of data analysis algorithms and neural network models can achieve high-quality and accurate detection of target physiological parameters, enabling real-time analysis of sensing data and direct visualization of diagnostic analysis. These learning and analysis abilities of ML have a positive impact on the processing of physiological signals. The wearable CGM system developed by Yang, as mentioned earlier, has long-term blood glucose level data for individual users. It is not difficult to imagine that by combining this data with machine learning algorithms, we can establish a prediction model. When blood glucose levels are unbalanced, we can remind users in advance to consume appropriate food or adjust drug doses. We can also use long-term data accumulation and analysis to develop more accurate diet and exercise plans for patients, in order to better control blood glucose levels.

3. Intrabiological drug monitoring

Monitoring the concentration of therapeutic drugs in living organisms is an important link in drug research and clinical application. The biosensor based on MNs can achieve high sensitivity, specificity, accuracy, and precision in monitoring drug concentration in living organisms. Rawson et al. reported the first human evaluation of real-time phenoxymethylpenicillin monitoring using a minimally invasive MN-based β-lactam biosensor in healthy volunteers [170]. The results showed that the MN biosensor was highly consistent with microdialysis in the PK spectrum of phenoxymethylpenicillin and determination of drug concentration in extracellular fluid, providing a proof of concept for its application in wider clinical practice.

Currently, the MN biosensors used for monitoring the concentration of therapeutic drugs in living organisms mainly utilize electrochemical conversion. Gowers et al. developed a MN-based potentiometer biosensor, which is coated with a pH-sensitive iridium oxide layer that can detect local pH β changes caused by the hydrolysis of β-lactamases fixed on the electrode surface, for continuous monitoring of β-lactam antibiotic concentrations in healthy human volunteers [171]. For user-friendly use of MN biosensors for drug monitoring in living organisms, machine learning models can be combined to predict drug concentrations in the body using experimental sensor data. Kadian et al. developed a wireless MNA integrated screen printed electrode electrochemical bedside device that supports machine learning for rapid and effective detection of lidocaine (Figure 14c) [172]. The manufactured sensor utilizes a novel ultra sharp MNA, which includes a reservoir at the bottom. By combining the MNA installed in the reservoir with paper-based microfluidic channels and capillary systems, as well as screen printed electrodes, it is used for on-site detection of lidocaine in tissue fluid. Derived from the peak current value and corresponding lidocaine concentration value obtained from the square wave voltammetry spectrum as the dataset, a linear regression model is used to calculate and analyze the dataset, with the peak current value as the input value and the lidocaine concentration as the output variable. Used to predict the concentration of lidocaine, and finally deployed the trained machine learning model into a web application for digital visualization of lidocaine concentration, encouraging on-site and user-friendly detection of lidocaine.

4. Freshness monitoring of meat

As an important food source, meat’s freshness directly determines its food safety. MNs have been integrated with smart responsive materials to detect various analytes. Specifically, MNs with visual responsive strategies can be applied to the field of meat freshness detection for the easy operation and convenience of customers.

Wang et al. designed a colorimetric MN sensor made of an edible hydrogel containing pH-responsive anthocyanins (Figure 14d) [173]. When the meat goes bad from fresh, the pH value of tissue fluid rises, resulting in the corresponding color change of MN patches. In order to achieve more convenient and accurate detection of meat freshness, the research team applied a deep learning algorithm to integrate with MN patches to form a smartphone application. The images of 2921 colorimetric MN patches with different freshness attached to meat were imported into a CNN model as the training source. By convoluting the color characteristics of MN patches, the meat freshness divided into “fresh”, “not fresh”, and “deteriorated” was output, and the accuracy was about 95.3%. With the addition of CNN, the application enables users to identify the freshness of meat in a fast, accurate, portable, and universal way through stored photos or real-time images of chicken and pork labeled by colorimetric MN patches. In addition, Kadian et al. developed a simple and low-cost MN colorimetric pH-sensing patch based on machine learning, which can be used for food quality detection (Figure 14e) [174]. Three-dimensional printed ultra sharp open side channel MNAs facilitate the autonomous extraction of fluids and the transport of colorimetric pH sensing through surface tension. The research team used multiple smartphones and different lighting conditions, including white, yellow, and a combination of the two, to create such an image dataset representing different pH values, used CNN to classify and learn the image dataset, and designed a simple web application to display the output results. Wound healing can also be reflected by pH value, so the colorimetric MN patch can also be applied to wound monitoring.

Based on the above application of machine learning-assisted MN biosensors, we can conclude that MN biosensors play a role in sensing and detection, while machine learning plays a role in data analysis. MN biosensors have multiple advantages. 1. MN biosensors can provide a more painless and comfortable sampling method than traditional blood collection, improving patient acceptance. 2. The non-invasive method of MN sensors can reduce damage and contamination to the sample. 3. As the sensing area is a small area at the tip of the sensor, the sensitivity of the MN structure to environmental disturbances is much lower. 4. MNs can accommodate multiple independent sensors on a single small array to provide timely multiplexing of molecular information. 5. MN biosensors have a small volume and are easy to carry. The introduction of machine learning improves the reliability and practicality of MN biosensors, making them more efficient and accurate. Even machine learning can combine MN biosensor data with other daily human data to achieve personalized health management. From this, we can infer that machine learning combined with MN biosensors will be applied to blood glucose monitoring, electrocardiogram monitoring, disease management, environmental detection and other aspects, with many practical and economic benefits.

## 5. Conclusions

This article first introduces the common types and basic principles of ML, followed by a detailed investigation of MN dimensions, structures, and preparation methods. By synthesizing existing literature, we have found that ML algorithms have shown great potential in MN design, especially in optimizing its performance by material selection and tuning the geometry. Specifically, ML has been employed to reduce pain when MNs penetrate [44], accelerate the material screening process [62], increase the maximum volume flow rate [46], and enhance the production efficiency and quality of MNs through 3D printing techniques [84]. These optimizations and simulations not only improve the patient’s comfort, but also significantly shorten the development cycle of MNs and therefore reduce resource consumption. Subsequently, we have introduced the wide application of MN technology in the field of disease treatment and sensing under different working principles and characteristics. In the field of treatment, MNs have been widely used in skin diseases, wound healing, hair loss treatment, oral health, and ophthalmology treatment due to targeted and painless drug delivery. ML can help to screen and discover the optimal drugs [144,145] delivered through microneedles. It can also assist in the prediction of drug penetration and diffusion through MNs [146,147], displaying its critical role in disease treatment. In terms of sensing, MNs can collect biomarkers in interstitial fluid of the skin in a minimally invasive manner, providing valuable sample resources for biosensor analysis. In recent years, the rise of MN biosensors, combined with the efficient data processing capabilities of ML, has achieved diversified application scenarios including wound health monitoring [163], physiological signal measurement [169], in vivo drug concentration monitoring [172], and meat freshness detection [173,174], demonstrating powerful real-time detection and analysis capabilities.

The integration of MN platforms and ML technology has been accelerated, which benefits the efficiency in MN design and production as well as the application of MNs.

## 6. Future Perspective

In recent years, MN has drawn tremendous attention as a type of miniaturized medical device. At the same time, the current research on ML technology is unprecedentedly prosperous. Through in-depth analysis of existing literature, we clearly realize that the integration of ML technology and MN platforms not only injects a strong impetus into the design and application of MNs, but also represents a key direction and milestone in the field of biomedical engineering.

In the future, the deeper integration of ML and MN technology will open up more innovative possibilities. As MN technology continues to develop, it will be able to generate richer, higher-quality data sets. These data sets will become valuable resources for training machine learning models, significantly improving the model’s prediction accuracy and generalization capabilities, thereby accelerating the development pace of ML in MN design optimization and diversified applications. At the same time, the continuous optimization and innovation of ML algorithms will be able to process more complex and diversified data, extract more critical and valuable features, and therefore accelerate the design and application of MN.

Specifically, in terms of MN design, more complex and varied geometry and material combination can be explored in the future, aiming to further improve MN penetration performance and patient comfort. Combined with advanced technologies such as deep learning, it is expected to achieve more accurate MN function prediction and performance evaluation, providing strong technical support for the design, sterilization, and storage of MNs as well as their applications in drug delivery and disease treatment. Moreover, with the development in wearable sensing materials for personal healthcare in recent years [175,176,177,178], wearable smart sensing devices that combine ML with MN technology have the potential for real-time monitoring of physiological indicators and precise and personalized drug administration. The combination of ML and MN will greatly improve the efficiency and accuracy of disease prevention, diagnosis, and treatment, thus bringing revolutionary changes to the health and clinical fields.

## Data Availability

Not applicable.

## Abbreviations

**Abbreviation**	**Definition**	**Abbreviation**	**Definition**
MN	Microneedle	CLIP	Continuous liquid interface production
SVM	Support vector machine	CAD	Computer-aided design
PCA	Principal component analysis	MNA	Microneedle array
ANN	Artificial neural network	TCD	TA-encapsulated candlelit-dissolving microneedle
LSTM	Long short-term memory	AGA	Androgenic alopecia
CNN	Convolutional neural network	FRM	Fractional radiofrequency microneedle
RNN	Recurrent neural network	DMN	Dissolving microneedle
GRU	Gated recurrent unit	AZA	Azelaic acid
GNN	Graph neural network	MP	Microsphere
GCN	Graph convolutional network	BP	Black phosphorus
GAT	Graph attention network	NIR	Near-infrared
NLP	Natural language processing	GT	Gelatin
VQA	Visual question answering	MTX	Methotrexate
MoE	Mixture of expert	ROS	Reactive oxygen species
MSE	Mean squared error	EGCG	Epigallocatechin gallate
ISF	Interstitial fluid	SN	Silicate nanosheet
VFR	Volumetric flow rat	GelMA	Gelatin methacryloyl
RF	Random forest	PTH	Parathyroid hormone
GBoost	Gradient boosting	PBN	Prussian blue nanoenzyme
GAT	Graph attention network	VEGF	Vascular endothelial growth factor
MEMS	Microelectromechanical system	SPM	Self-plugging microneedle
TPP	Two-photon polymerization	RA	Rheumatoid arthritis
FDM	fused deposition modeling	TSA	Trichostatin A
SLA	Stereolithography	HDAC4	Histone deacetylase 4
DLP	Digital light processing	MLR	Multiple linear regression
PBP	Projection printing	PVA	Polyvinyl alcohol
LCD	Liquid crystal display	CGM	Continuous glucose monitoring
